# Long-term storage limits PCR-based analyses of malaria parasites in archival dried blood spots

**DOI:** 10.1186/1475-2875-11-339

**Published:** 2012-10-08

**Authors:** Joyce Hwang, Juthamas Jaroensuk, Mara L Leimanis, Bruce Russell, Rose McGready, Nicholas Day, George Snounou, Francois Nosten, Mallika Imwong

**Affiliations:** 1 Shoklo Malaria Research Unit, Mae Sot, Tak Province, Thailand; 2 Singapore Immunology Network, Biopolis, Agency for Science Technology and Research (A*STAR), Singapore, Singapore; 3 Department of Molecular Tropical Medicine and Genetics, Faculty of Tropical Medicine, Mahidol University, Bangkok, Thailand; 4 Centre for Vaccinology and Tropical Medicine, Churchill Hospital, Oxford, UK; 5 INSERM UMR S 945, Paris F-75013, France; 6 Université Paris 6, Pierre & Marie Curie, Faculté de Médecine Pitié-Salpêtrière, Paris, France; 7 Center for Emerging and Neglected Infectious Diseases, Mahidol University, Bangkok, Thailand

**Keywords:** Archival blood spots, Plasmodium falciparum, Polymerase chain reaction

## Abstract

**Background:**

Blood samples collected in epidemiological and clinical investigations and then stored, often at room temperature, as blood spots dried on a filter paper have become one of the most popular source of material for further molecular analyses of malaria parasites. The dried blood spots are often archived so that they can be used for further retrospective investigations of parasite prevalence, or as new genetic markers come to the fore. However, the suitability of the template obtained from dried blood spots that have been stored for long periods for DNA amplification is not known.

**Methods:**

DNA from 267 archived blood spots collected over a period of 12 years from persons with microscopically confirmed *Plasmodium falciparum* infection was purified by one of two methods, Chelex and Qiagen columns. These templates were subjected to highly sensitive nested PCR amplification targeting three parasite loci that differ in length and/or copy number.

**Results:**

When a 1.6 kb fragment of the parasites’ small subunit ribosomal RNA was targeted (primary amplification), the efficiency of *P. falciparum* detection decreased in samples archived for more than six years, reaching very low levels for those stored for more than 10 years. Positive amplification was generally obtained more often with Qiagen-extracted templates. *P. falciparum* could be detected in 32 of the 40 negative Qiagen-extracted templates when a microsatellite of about 180 bp was targeted. The remaining eight samples gave a positive amplification when a small region of 238 bp of the higher copy number (20 to 200) mitochondrial genome was targeted.

**Conclusions:**

The average length of DNA fragments that can be recovered from dried blood spots decreases with storage time. Recovery of the DNA is somewhat improved, especially in older samples, by the use of a commercial DNA purification column, but targets larger than 1.5 kb are unlikely to be present 10 years after the initial blood collection, when the average length of the DNA fragments present is likely to be around a few hundred bp. In conclusion, the utility of archived dried blood spots for molecular analyses decreases with storage time.

## Background

The development of methods to amplify specific segments of DNA, of which the earliest and most commonly used is the polymerase chain reaction (PCR), has made it possible for the first time to conduct detailed molecular investigations of malaria parasites from a few microlitres of infected blood. Indeed, the specificity and sensitivity of PCR are such that DNA fragments can be routinely amplified from a blood aliquot that contains a few parasites only. Finger-prick samples, the simplest and least invasive sampling procedure, which hitherto served almost exclusively to generate blood smears for microscopic examination, thus became the collection method of choice for molecular studies of *Plasmodium* parasites based on PCR amplification. The demonstration that successful PCR analyses can be performed from DNA extracted from few drops of bloods dried on a filter paper that have been kept and shipped at room temperature [1] revolutionized molecular epidemiological studies of malaria. Typically 20 μl to 50 μl of blood are obtained and blotted directly on a filter paper that is then left to dry before being individually sealed with an inert desiccant in a plastic bag. Given the ease of collection and that no cold chain is needed, this procedure has been almost universally adopted for field-based investigations. Moreover, dried blood spots can be stored in a fridge or even at room temperature until DNA extraction. The most common uses for this DNA are: parasite detection and identification, investigation of parasite population structure based on the genetic diversity of selected loci, analysis of mutations associated with drug resistance, and finally *Plasmodium* genotyping to correct outcome of *in vivo* drug trials in endemic settings.

It has now become customary to include dried blood spots collection in many trials and field surveys, and after the primary aims of the study have been achieved, duplicate spots are often retained as archival material, usually stored at room temperature. These dried blood spot collections afford an opportunity to conduct retrospective molecular epidemiological investigations on malaria parasites. There is evidence to suggest that the suitability of DNA obtained from dried blood spots may be affected not only by the extraction method, but also by the type of filter paper used as well as the storage temperature and duration [2-4]. In these few studies that have examined the effect of such long-term storage on the efficacy of DNA amplification, the results were somewhat contradictory. A loss of PCR sensitivity was observed in two studies [2,4], while some improvement in this sensitivity was noted in the third [3].

Over the years, researchers at the Shoklo Malaria Research Unit (SMRU) amassed a substantial collection of dried blood spots collected from patients recruited to a wide spectrum of clinical, chemotherapeutic and epidemiological investigations on malaria in populations in and around Mae Sot, a large town on the Thai-Myanmar border. The epidemiology of malaria parasites has changed considerably during this period. For example, *Plasmodium falciparum* is no longer the dominant species, and the pattern of resistance to various anti-malarial drugs has shifted such that *P. falciparum* parasites are now resistant even to artemisinin derivatives therapy (ACT) [5] and resistance to chloroquine is emerging in *Plasmodium vivax*[6]. Concurrently, technical advances have led to a massive increase in sequence data from malaria parasites. The complete genomes of *P. falciparum* and *P. vivax* are now available [7,8], as are extensive sequence data from an increasing number of different strains from these two species [9,10]. This has opened the way to assess the significance of genetic polymorphisms predicted or found associated to drug resistance, as was the case for resistance to the artemisinins [11], clinical evolution or the acquisition of immunity. In this context, the dried blood spots collected over the years represent a precious parasite DNA repository [5,11].

The dried blood spots have been stored at ambient temperature under normal atmospheric conditions. Thus, in order to ensure that they could serve as a reliable source of *Plasmodium* DNA for further investigations, it was necessary to establish whether storage has led to a loss in the ability to amplify specific DNA fragments by PCR. To address this issue, subsets of dried blood spots collected over 12 years starting from 1998 from patients diagnosed with *P. falciparum* that were recruited to various randomized trials were tested.

## Methods

### Dried blood spots

Blood samples dried on filter paper were collected from febrile patients presenting at clinics of the Shoklo Malaria Research Unit (SMRU) and screened as part of treatment trials that were conducted between 1998 and 2009 and approved by the Oxford University Tropical Research Ethics Committee (OXTREC) and the Faculty of Tropical Medicine, Mahidol University Ethics Committee. All the samples were obtained from non-pregnant patients diagnosed with *P. falciparum* by microscopic examination of thick and thin Giemsa-stained blood films. Parasite densities were estimated by enumeration of parasites per 500 white blood cells (WBC) or 1,000 red blood cells (RBC), in thick and thin films, respectively. Multiple blood spots (usually 3) were obtained by spotting approximately 30 μl of finger-prick blood onto Whatman 3MM filter papers (Maidstone, UK). These blood spots were then maintained away from insects and sunlight under dry conditions until arrival from the clinics to the laboratory at SMRU, where they were air dried over night and stored usually with silica gel absorbent at room temperature in plastic bags until DNA extraction.

### DNA extraction and PCR protocols

DNA was extracted from the blood spots by the Chelex method [1] or using the QIAamp DNA mini kit (Qiagen) according to the manufacturer’s instructions. Approximately 30 μl of blood were used per filter spot. The final template volume obtained after Chelex extraction was 125 μl, and that obtained after QIAamp extraction was 150 μl. The presence of *P. falciparum* was assayed by one or more of three PCR assays; throughout numerous negative controls were included to ensure lack of contamination.

1) Nested PCR targeting the small subunit ribosomal RNA (ssrRNA) genes was conducted as described previously [12] with minor modifications. Briefly 4 μl of the template DNA (equivalent to 0.96 μl and 0.8 μl of blood for the Chelex-extracted and QIAamp-extracted spots, respectively) were used in a primary amplification reaction with the genus-specific oligonucleotide primers rPLU1 and rPLU5 (expected product of 1.6 kb to 1.7 kb, there are five genes in the parasite’s genome), and the product served as a template for a secondary amplification reaction using the species-specific oligonucleotide primers rFAL1 and rFAL2 (expected product size 206 bp). The reactions were carried out in a total volume of 20 μl using Taq polymerase (Bioline) at a final concentration of 5 u/100 μl and in the presence of 5 mM MgCl_2_, 500 μM dNTPs, and 250 nM of each primer. After an initial denaturation at 95°C for 5 min, 24 amplification cycles consisting of 2 min at 58°C (annealing), 2 min at 72°C (extension) and 1 min at 94°C (denaturation) were carried out. Following a final annealing step and an extension step of 5 min, the reaction mix was cooled to room temperature and then stored at 4°C. The secondary amplification reaction was initiated using 1 μl of the product of the primary reaction, under the same conditions except that 29 cycles of amplifications were carried out. The presence of an amplified fragment was ascertained by electrophoresis of 10 μl of the product on a 2% agarose gel in the presence of ethidium bromide that allows visualising the DNA under UV trans-illumination.

2) A semi-nested PCR targeting the *P. falciparum* TAA87 microsatellite [13] was conducted as described above except that the oligonucleotide primers TAA87-3F and TAA87-R were used for the primary amplification reaction (expected product of about 180 bp), and the product served as a template for a secondary amplification reaction using the oligonucleotide primers TAA87-F and TAA87-R (expected product size 93 bp - 114 bp). The reactions were carried out in a total volume of 20 μl using 0.4 u of Taq polymerase (Bioline) in the presence of 2.5 mM MgCl_2_, 125 μM dNTPs, and 250 nM of each primer. After an initial denaturation at 95°C for 5 min, 24 amplification cycles consisting of 2 min at 52°C (annealing), 2 min at 72°C (extension) and 1 min at 94°C (denaturation) were carried out. Following a final annealing step and an extension step of 5 min, the reaction mix was cooled to room temperature and then stored at 4°C. The secondary amplification reaction was initiated using 1 μl of the product of the primary reaction, under the same conditions except that 29 cycles of amplifications were carried out. The presence of an amplified fragment was ascertained by electrophoresis of 10 μl of the product on a 2% agarose gel in the presence of ethidium bromide that allows visualising the DNA under UV trans-illumination.

3) A semi-nested PCR targeting a fragment on the mitochondrial genome, with the primary reaction using the oligonucleotide primers UM-F4 + UM-OR4 to give an expected product size of 238 bp, and a secondary reaction using UM-F4 + UM-NR4 to give an expected product size of 108 bp (UM-F4 5’-AAAGGAACTCGACTGGCCTA, UM-OR4 5’-CCAAATAAAAATGAAAACCATAAA, and UM-NR4 5’-ATACAGTCCCAGCGACAGC). The reactions were carried out using 4 μl of the DNA template in a total volume of 20 μl using 0.4 u of Taq polymerase (Bioline) in the presence of 3 mM MgCl_2_, 125 μM dNTPs, and 250 nM of each primer. After an initial denaturation at 95°C for 5 min, 24 amplification cycles consisting of 1 min at 55°C (annealing), 1 min at 7°C (extension) and 1 min at 94°C (denaturation) were carried out. Following a final annealing step and an extension step of 5 min, the reaction mix was cooled to room temperature and then stored at 4°C. The secondary amplification reaction was initiated using 1 μl of the product of the primary reaction, under the same conditions except that 29 cycles of amplifications were carried out. The presence of an amplified fragment was ascertained by electrophoresis of 10 μl of the product on a 2% agarose gel in the presence of ethidium bromide that allows visualising the DNA under UV trans-illumination.

In order to establish that the sensitivity of the amplification reactions was optimal for the different amplification assays, quality control templates were purified using the QIAamp DNA mini kit from *P. falciparum* patient samples, for which the parasitaemia (parasites/μl) was determined with accuracy by microscopic examination, that were diluted serially with whole blood (with the resulting parasitaemia confirmed by blood smears prepared from the resulting mixtures). The templates used as positive controls that were included in all the amplification runs conducted during the course of this study, always included one obtained by the highest dilution that consistently gave a positive amplification results (usually 1–10 P/μl).

### Statistical analysis

Statistical analyses were performed using GraphPad Prism 4 software (version 4).

## Results

**Figure 1:**
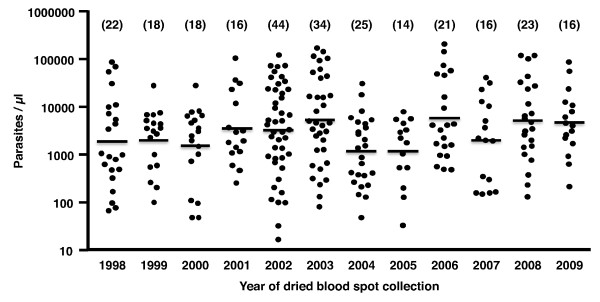
**The range of the parasitaemias recorded by microscopy for the dried blood spots used in this study.** The number of samples for each year is recorded in brackets above the data for each year.

A total of 267 dried blood spots were selected for the study. All were selected from sample sets obtained between 1998 and 2009 (range of 18 to 44 samples per year, Figure 1). All 267 were obtained from patients with microscopically confirmed *P. falciparum* at the time of collection. For the subsets from each year the range of parasitaemia represented was >250 P/μl to <25,000 P/μl (Figure 1). Thus, it would be expected that a sensitive PCR assay would detect *P. falciparum* in all the samples. The study was initiated in 2010, thus the dried filter spots from 2009 had already been in storage for about one year.

### Influence of the template preparation method

Although blood dried on filters provides the most practical way to collect and store field samples, extraction of the DNA to serve as a suitable template for PCR represents a time-consuming, potentially costly, step. Over the years, different protocols, with various efficiencies at yielding suitable templates [4] have been tested. The use of the first protocol published to do so, extraction with Chelex [1], is still in common usage because it is relatively cheap though the DNA obtained is relatively impure. Commercial kits, such as Qiagen’s QIAamp DNA mini kit, that yield highly purified DNA from dried filter paper are now widely available, but they are relatively costly.

**Figure 2:**
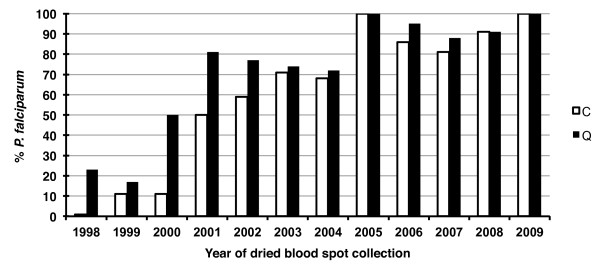
**Efficiency of the PCR assay based on the ssrRNA genes when applied to template purified by Chelex (C) *vs *Qiagen (Q) from dried blood spots collected between 1998 and 2009 that were confirmed by microscopy to be positive for *Plasmodium falciparum*.**

In a first instance, duplicate dried blood spots were used to obtain template, by Chelex extraction from one or by QIAamp DNA mini kit from the other punch. A standard specific and sensitive nested PCR for the detection of *P. falciparum*, based on the ssrRNA S-type gene [12], was then applied in parallel to the two sets of templates. Using DNA obtained from *P. falciparum*-infected blood serially diluted with whole blood, the sensitivity of the nested PCR assay was determined to be close to the absolute maximum, i.e. 1–10 parasites/aliquot assayed. The results of the archival dried blood spots assays are graphically summarized in Figure 2. First, the ability to detect the *P. falciparum* parasites present in the blood on the day of collection decreased with the blood spots’ period of storage. Thus, less than 50% of the samples collected before 2001 yielded a positive amplification, i.e. those stored for nine years or more. Second, both extraction methods were equally effective in producing template yielding positive amplification for samples from 2003 onwards, i.e. those stored for seven years or less. However, for the older blood spots, i.e. those stored for eight years or more, templates purified by the Qiagen kit yielded substantially more positive amplification than those purified by the Chelex method.

For 12 samples, the template purified by Chelex was positive but not that purified by Qiagen. In half of these, the parasitaemia was below 500 P/μl, and three of the four samples with the highest parasitaemia (3,360 P/μl–6,640 P/μl) were collected more than 10 years ago. This is likely to be due to the “all-or-none” effect of methods where samples with target numbers in the aliquot assayed are close to limits of the assay’s sensitivity.

The overall ability to detect *P. falciparum* by the standard nested PCR protocol based on ssrRNA genes decreases as the dried blood spots are stored beyond five years regardless of the extraction method used (Figure 2). A minor proportion of the blood spots stored beyond 10 years yielded positive amplification, though sensitivity was improved when the template was purified using a Qiagen kit (Figure 2). The presence of an amplification reaction inhibitor, maybe in concentrations increasing with period of storage, was assessed using a subset of templates purified by both methods (Chelex and Qiagen) from older and more recent dried blood spots. This was achieved by spiking the assay for each template with an aliquot of purified *P. falciparum* genomic DNA that is close to the detection limit of the nested PCR assay. No evidence of inhibition could be obtained in either set of templates (data not shown). Therefore, the loss of sensitivity with increasing storage period is due to a loss of template.

### Influence of the amplification target

A reduction in the number of amplifiable targets in the templates purified from the dried blood spots must be due to DNA degradation, most likely random breakage and/or loss of residues (for e g, depurination). Both types of DNA damage would in a first instance lead to an effective shortening of the average length of the DNA fragments present, and eventually in a loss of the DNA altogether. No published investigations that document the rate at which this might occur could be found.

The loss in detection sensitivity resulting from a shortening of the average length of the template DNA molecules would increase with the length of the fragment targeted for amplification. The primers targeting the ssrRNA gene in the primary amplification reaction amplify a fragment of about 1,600 bp (206 bp for the secondary reaction). Thus, it was hypothesized that relatively large size of the target fragment might account for the loss of sensitivity documented above. This view is consistent with the improved PCR detection recorded when the template was prepared from the dried blood spots by chemical lysis followed by membrane binding/elution (the principle of the Qiagen kit), relative to that observed after boiling (the central step of the Chelex method), a treatment known to lead to DNA degradation [14].

**Figure 3:**
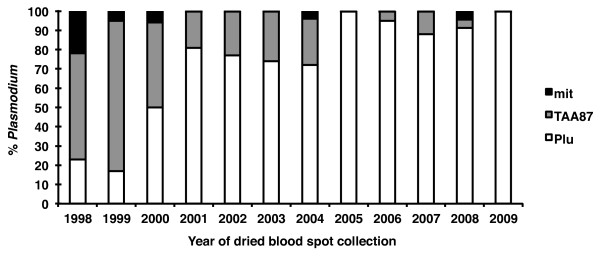
**Cumulative proportion of *Plasmodium falciparum *and *Plasmodium *positive dried blood spots using the PCR assays targeting the ssrRNA genes (Plu) for all 267 samples or a microsatellite (TAA87) for 40 samples, or the mitochondrial genome (mit) for eight samples, respectively.** All assays were performed on templates prepared by QIAamp extraction.

In order to ascertain whether a reduction of average fragment length played a role in the loss of parasite detection sensitivity, another PCR assay where the target size is relatively small, a *P. falciparum*-specific microsatellite (TAA87 with an expected size of about 180 bp for the first reaction and 100 bp for the second) was used. Given the poor results obtained with Chelex-purified templates, all further PCR assays were carried out using the Qiagen kit-purified templates. In a first instance, the sensitivity of the amplification targeting the TAA87 microsatellite was determined to be also close to the absolute maximum, i.e. 1–10 parasites/aliquot tested. It was not felt necessary to carry out the assay on the templates for all 267 samples; nonetheless, it was validated on the 14 PCR positive samples collected in 2005 (range 32 P/μl to 7,520 P/μl) in which it correctly identified all as positive for *P. falciparum*. The results for the TAA87 microsatellite amplification assays carried out on all the Qiagen-purified templates that were negative by the nested ssrRNA PCR targeting the ssrRNA (n = 40) are presented in Figure 3. Nearly all these templates yielded a positive amplification, though eight remained negative (five collected in 1998, and one collected in 2000, 2004 or 2008).

The number of parasites (circa 5,000 P/μl or less) and age of the dried blood spots (five to 11 years) was consistent with a loss in sensitivity in seven of these samples, but not the last that was collected in 2008 from a blood sample with nearly 24,000 P/μl, making it the only sample with an unexpected discordance between microscopy and PCR was observed. Whether this discordance was due to microscopic misdiagnosis, sample mislabelling or mishandling is not known.

Given that reducing the amplification target size significantly improved *P. falciparum* detection sensitivity, it was hypothesized that targeting a similarly small fragment for amplification also present in multiple copies in the genome might increase sensitivity even further. The copy number of the mitochondrial genome (6 kb) is considered to be 20 to 200 [15], a wide range that might reflect copy number increases as the erythrocytic parasites mature. This approach has been used previously and shown to improve detection sensitivity [16]. A set of genus-specific oligonucleotides, i.e. specific to the mitochondrial genome of any species of *Plasmodium*, was designed for nested amplification of small fragments (238 bp for the primary reaction and 108 bp for the secondary reaction). The assay was shown to be more sensitive in detecting parasites than the ssrRNA- and microsatellite-based assays, since it was capable of detecting 0.1 parasites per aliquot routinely, and gave a positive amplification when tested on a subset set of the templates obtained from dried blood spots of low parasitaemia that were positive for the ssrRNA and/or the TAA87 microsatellite amplification assays (data not shown). All eight samples that had remained negative for the ssrRNA and TAA87 microsatellite PCR protocols were found to be positive when assayed by the mitochondrial amplification protocol (Figure 3). Given that the mitochondrial assay detects all *Plasmodium* species, it is possible that these samples were negative with the *P. falciparum*-specific microsatellite assay because the samples contained another species, such as *P. vivax* a highly prevalent parasite species in Thailand, that was microscopically misdiagnosed as *P. falciparum*.

## Conclusions

The simplicity, practicability and low-cost of the collection, transport and storage of blood samples as dried spots on filter paper has significantly contributed to disseminate the molecular analysis of *Plasmodium* parasites in endemic countries. Prior to this, it was considered necessary to establish a cold chain from the time the samples were collected in the field to the moment they were processed for DNA extraction, often after storage and transport to distant laboratories. The dried blood spot obviated this, and by the end of the 1990s it had rapidly become the method of choice for sample collection in most of the epidemiological investigations and field trials conducted globally by the malaria community. These samples occupy a relatively small volume and can be stored at room temperature, moreover only part of the blood spot or one from sets collected in duplicate sets were needed for the purposes of the investigations. Thus, it is likely that large collection of archived blood spots can still be found in various institutions in and outside endemic countries. This represents a rich, yet cheap and easily accessible, source of material for retrospective molecular analysis of *Plasmodium* parasites.

However, the data presented here indicates that the suitability of the DNA that could be extracted from archived blood spots for such molecular analyses diminishes with time, most probably because of degradation leading to the fragmentation of DNA. It is likely that few of the DNA molecules that can be extracted after 10 years of storage would be larger than a few hundred base pairs. This would restrict the analysis of genetic diversity to haplotypes that do not exceed this size, and parasite detection to amplification protocols that target small amplicons. It should be noted that these conclusions are based on blood samples that were dried on a single type of filter paper (Whatman 3MM) and stored at room temperature. Desiccant (silica gel or a proprietary bag) was included in each sealed plastic bag containing the dried blood spots, but it was not replaced regularly with a fresh batch, thus, it is unlikely that it would have offered protection from humidity beyond a few weeks after collection. The degradation of DNA is enhanced at higher temperatures and as humidity increases. These two variables are rarely controlled when it comes to the storage of dried blood samples by researchers, and they are likely to play a significant role in the loss of sensitivity for samples stored in tropical and subtropical climates. Nonetheless, in our experience a very large majority of our colleagues store their archival dried blood spots, like us, under sub-optimal conditions. It is possible that DNA integrity could be maintained for longer durations when the dried blood spots are stored under controlled conditions where humidity is maintained at low levels and in the cold, though this would substantially increase the cost of archiving. Alternatively, the use of specialized filters impregnated with chemicals that protect DNA, such as Whatman FTA™ cards or their equivalent, should be preferred despite their increased cost because the DNA should be protected from degradation. Our conclusions might not apply to samples stored under these optimal conditions. Ultimately, it would be desirable to propose a standardized method for the collection of dried blood spots and their storage. The additional costs are justifiable because it is probable that the samples collected today would be of great value for future studies.

## Competing interests

The authors declare that they have no competing interests.

## Authors’ contributions

GS, MI, BR, RM, and FN designed the study. RM and FN carried out sample collections. JH, JJ and MI processed the samples and conducted the PCR samples and helped with data collection and management. JH, ML, BR, RM, ND, GS, FN and MI analysed the data. JH, ML, MI and GS drafted the manuscript. All authors read and approved the final manuscript.
